# Design and preclinical evaluation of a ^99m^Tc-labelled diabody of mAb J591 for SPECT imaging of prostate-specific membrane antigen (PSMA)

**DOI:** 10.1186/2191-219X-4-13

**Published:** 2014-03-07

**Authors:** Florian Kampmeier, Jennifer D Williams, John Maher, Gregory E Mullen, Philip J Blower

**Affiliations:** 1 Division of Imaging Sciences and Biomedical Engineering, King's College London, 4th Floor Lambeth Wing, St. Thomas' Hospital, London SE1 7EH, UK; 2 Department of Research Oncology, King's Health Partners Integrated Cancer Centre, King's College London, Guy's Hospital Campus, Great Maze Pond, London SE1 9RT, UK; 3 Department of Immunology, Barnet and Chase Farm NHS Trust, Barnet, Hertfordshire EN5 3DJ, UK; 4 Department of Clinical Immunology and Allergy, King's College Hospital NHS Foundation Trust, Denmark Hill, London SE5 9RS, UK; 5 Division of Chemistry, King's College London, Britannia House, 7 Trinity St, London SE1 1DB, UK

**Keywords:** Prostate carcinoma (PCa), PSMA, Diabody, Single photon emission computed tomography (SPECT), ^99m^Tc, Molecular imaging

## Abstract

**Background:**

Sensitive and specific detection of nodal status, sites of metastases and low-volume recurrent disease could greatly improve management of patients with advanced prostate cancer. Prostate-specific membrane antigen (PSMA) is a well-established marker for prostate carcinoma with increased levels of expression in high-grade, hormone-refractory and metastatic disease. The monoclonal antibody (mAb) J591 is directed against an extracellular epitope of PSMA and has been shown to efficiently target disseminated disease including metastases in lymph nodes and bone. Its use as a diagnostic imaging agent however is limited due to its slow pharmacokinetics. In this study a diabody derived from mAb J591 was developed as a single photon emission computed tomography (SPECT) tracer with improved pharmacokinetics for the detection of PSMA expression in prostate cancer.

**Methods:**

A diabody in V_H_-V_L_ orientation and with a C-terminal cysteine was expressed in HEK293T cells and purified by a combination of metal ion affinity and size exclusion chromatography. Specificity and affinity were determined in cell binding studies. For SPECT imaging, the diabody was site-specifically labelled with [^99m^Tc(CO)_3_]^+^ via the C-terminal His tag and evaluated in a subcutaneous DU145/DU145-PSMA prostate carcinoma xenograft model.

**Results:**

J591C diabody binds to PSMA-expressing cells with low nanomolar affinity (3.3 ± 0.2 nM). SPECT studies allowed imaging of tumour xenografts with high contrast from 4 h post injection (p.i.). *Ex vivo* biodistribution studies showed peak tumour uptake of the tracer of 12.1% ± 1.7% injected dose (ID)/g at 8 h p.i. with a tumour to blood ratio of 8.0. Uptake in PSMA-negative tumours was significantly lower with 6.3% ± 0.5% at 8 h p.i. (*p* < 0.001).

**Conclusion:**

The presented diabody has favourable properties required to warrant its further development for antibody-based imaging of PSMA expression in prostate cancer, including PSMA-specific uptake, favourable pharmacokinetics compared to the parental antibody and efficient site-specific radiolabelling with ^99m^Tc.

## Background

Prostate cancer (PCa) has a good prognosis when localised to the prostate gland or when disease has not spread beyond the regional lymph nodes. However, the 5-year survival rate drops from 99% to approximately 27% in patients with evidence of distant metastases [1]. Management of patients with advanced prostate cancer could be greatly improved by more sensitive, non-invasive techniques that allow more accurate staging and localisation of sites of metastases and low-volume recurrent disease.

Prostate-specific membrane antigen (PSMA; also known as glutamate carboxypeptidase II (GPCII)) is a well-established marker for prostate carcinoma. Elevated expression of PSMA is found in virtually all prostate cancers with the highest levels found in high-grade, hormone-refractory and metastatic disease [2-6]. Expression of PSMA is also found in the neo-vasculature of solid tumours [7]. Expression in non-malignant tissue is found in the prostate epithelium and, to a limited extent, in brush border cells of the duodenum, kidney proximal tubules, breast epithelium, neuroendocrine cells in colonic crypts and in the brain [3,7,8]. Imaging studies with PSMA-specific small molecules in man have further shown accumulation in lacrimal and salivary glands [9,10]. This very restricted expression pattern makes PSMA an excellent target for detection and targeted therapy of prostate cancer. Several studies have demonstrated a correlation between increased PSMA expression and higher Gleason score. Although not validated as a predictive marker, it has been suggested that PSMA expression levels in the primary tumour can predict disease outcome [11,12].

Several antibodies that recognise extracellular epitopes of PSMA have been developed [13,14]. The monoclonal antibody (mAb) J591 was the first of these described, and the targeting properties of a de-immunised version have been characterised in several combined radioimmunotherapy and imaging clinical studies [15-19]. Despite the excellent targeting properties reported in these studies, imaging had to be performed several days after injection of the tracer in order to obtain images of sufficient contrast as a result of the long circulation time of the full-length antibody conjugate.

Antibodies can be engineered into smaller fragments that largely retain the original antigen binding properties but with more rapid pharmacokinetics, potentially enabling tracer injection and imaging on the same day. Diabodies with their intermediate size of approximately 55 kDa are likely to represent a good balance between circulation time/systemic clearance, target accumulation and tissue penetration; good contrast images can be obtained within 1 to 8 h in preclinical studies [20-22]. They consist only of the variable domains of an antibody connected by a short (typically five to eight amino acids) linker, which promotes the formation of a small, bivalent and homo-dimeric protein. Introduction of an additional cysteine at or near the C-terminus can result in the formation of an inter-chain disulfide bond that further stabilises the otherwise non-covalent dimer (Additional file 1). Diabodies against various targets have been engineered and radiolabelled for preclinical imaging studies, mainly using positron emission tomography (PET) [23-26].

^99m^Tc is an attractive choice for labelling intermediate-sized recombinant proteins such as diabodies for single photon emission computed tomography (SPECT), for several reasons. First, the tricarbonyl chemistry allows direct chelation of ^99m^Tc by genetically encoded tags, such as the (His)_6_-tag, which simplifies the labelling process [27]. Second, its half-life of 6 h matches well with the average serum half-life of diabodies. Third, it provides excellent image quality at low radiation doses compared to isotopes with a longer half-life such as ^111^In and ^89^Zr that are needed for imaging with whole antibodies.

Here we describe the development and preclinical evaluation of a diabody derived from the anti-PSMA antibody J591, site-specifically labelled with ^99m^Tc, for SPECT imaging of PSMA expression in prostate cancer.

## Methods

### Antibody construction, expression and purification

A single-chain fragment variable (scFv) of J591 in V_H_-V_L_ orientation was PCR-amplified from the SFG P28z vector [28], converting it into a diabody by shortening the linker to GGGGS using overlapping primers. The diabody was cloned into pSEC-tag2 (Invitrogen, Carlsbad, CA, USA)-based mammalian expression vector pMS-C with N-terminal Ig-kappa leader and a C-terminal (His)_6_-tag followed by a cysteine (J591Cdia). A diabody without C-terminal cysteine was generated to evaluate the effect of the additional cysteine. HEK293T cells were transfected with the expression vector using Lipofectamine 2000 (Life Technologies, Paisley, UK), and transfected cells were selected with 100 μg/ml Zeocin (Life Technologies) before expanding to triple flasks for protein production. The diabody was purified from HEK293T culture supernatant by Ni-NTA chromatography (5 mL Ni-NTA Superflow Cartridge, Qiagen, Manchester, UK) followed by a gel filtration step (Superdex 75 HR 10/30, GE-Healthcare, Little Chalfont, UK) using an AKTA FPLC system (GE-Healthcare). Purified protein (in phosphate-buffered saline (PBS; pH 7.0) for subsequent maleimide conjugation or PBS/500 mM NaCl, pH 7.4, for ^99m^Tc labelling) was concentrated to >1 mg/mL and stored in aliquots at −80°C. Purity was assessed by Coomassie staining after sodium dodecyl sulphate polyacrylamide gel electrophoresis (SDS-PAGE) and analytical size exclusion high-pressure liquid chromatography (HPLC) (BioSep SEC-s2000, Phenomenex, Cheshire, UK). Protein concentration was determined by UV absorption at 280 nm using the molar extinction coefficient of the diabody (*E*_280nm_, 51,130 M^−1^ cm^−1^; MW_monomer_, 27.18 kDa; determined with ProtParam [29]).

### Generation of PSMA-expressing DU145 cells

A synthetic DNA fragment that encodes for PSMA was synthesised by GenScript (Piscataway, NJ, USA). To allow cloning into SFG, the fragment consisted of bases 6137 to 6344 of the SFG P28z retroviral vector (includes a 5′ *Age*I site [28]), followed by a codon-optimised cDNA that encodes for full-length human PSMA and flanked by a 3′ *Xho*I site. This fragment was cloned as an *Age*I-*Xho*I fragment into SFG, replacing bases 6137 to 6344 of the vector. Retrovirus was packaged using PG13 cells (European Collection of Cell Cultures, Porton Down, UK). Transduction of DU145 cells (Cancer Research UK Organization) with SFG PSMA was performed using conditioned medium derived from PG13 cells. This was followed by immunoselection using mouse anti-human PSMA antibody (clone K0142-3, MBL International, Woburn, MA, USA) followed by collection of PSMA-expressing cells using sheep anti-mouse Ig-coated paramagnetic beads (Life Technologies, Paisley, UK).

### Cell culture

Human prostate carcinoma cells DU145, DU145-PSMA and LNCaP (LCG Standards, Bury, UK: CRL-1740) were cultured in RPMI 1640 supplemented with 10% FBS, 2 mM l-glutamine and penicillin/streptomycin (cell culture reagents and consumables were purchased from PAA, Somerset, UK). For induction of tumours, sub-confluent cells were washed with PBS, harvested by brief trypsinisation and re-suspended, after an additional washing step, in RPMI 1640 medium with 3.5 × 10^6^ cells in 50 to 80 μl.

### Fluorophore conjugation

Fluorophores were conjugated to the diabody via the C-terminal cysteines. The protein in PBS/2 mM EDTA (pH 7.0) was incubated for 30 min at 37°C with a 10-fold molar excess of TCEP (Bond-Braker, Pierce Biotechnology, Rockford, IL, USA) before addition of a 20-fold molar excess Alexa488-C_5_-maleimide (Life Technologies) for 2 h at room temperature (RT). Unconjugated fluorophore was removed by size exclusion chromatography using a Superdex 75 column, and the conjugated protein was concentrated using MWCO membrane spin filters (Vivaspin 2, MWCO 5000, Sartorius, Darmstadt, Germany).

### Flow cytometry

Saturation binding studies were carried out to analyse the *in vitro* binding properties of the diabody. PSMA^+^ or PSMA^−^ cells (4 × 10^5^) were incubated with serial dilutions of fluorescein- or Alexa488-labelled diabody in 250 μl PBS for 30 min on ice. Mean fluorescence values were determined by flow cytometry (FACSCalibur with Cellquest software, BD Biosciences, Oxford, UK), and the signal obtained with the highest concentration of J591Cdia-Alexa488 on DU145-PSMA cells was set as 100%. Data were analysed using a one-site total binding model (GraphPad Prism version 5.00 for Windows, GraphPad Software, San Diego, CA, USA).

### Confocal microscopy

Receptor-mediated internalisation of J591Cdia was analysed by confocal microscopy. DU145 or DU145-PSMA cells were seeded in chamber slides (Lab-Tek, Fisher Scientific, Loughborough, UK) and incubated when confluent with 4 μg/ml of J591Cdia-Alexa488 for 30 min at 4°C or 37°C. Nuclear counterstaining was achieved using 4′,6-diamidino-2-phenylindole (DAPI; ProLong Gold, Life Technologies, Paisley, UK). Pictures were taken with a TCS SP5 II confocal microscope (Leica, Milton Keynes, UK).

### ^99m^Tc radiolabelling

For imaging and biodistribution and cell binding studies, the diabody was labelled with ^99m^Tc-tricarbonyl ([^99m^Tc(CO)_3_]^+^) via the C-terminal (His)_6_-tag. The IsoLink kit (Covidien, Petten, The Netherlands) was used to convert 2,200 to 2,500 MBq of ^99m^Tc pertechnetate in 400 to 500 μl saline to [^99m^Tc(CO)_3_]^+^. After heating to 97°C for 30 min, the kit was neutralised with 1 M HCl and conversion to [^99m^Tc(CO)_3_]^+^ was verified by thin-layer chromatography (TLC; glass-backed silica gel 60, Merck, Darmstadt, Germany; mobile phase: 1% HCl in methanol). The diabody was incubated at 37°C with 5.5 MBq/μg for 1 h and passed through a G25 Minitrap column (GE-Healthcare, Little Chalfont, UK) to remove residual unbound ^99m^Tc and potential colloids. Labelling and final radiochemical purity were monitored by TLC in 60 mM citrate buffer, pH 5.5 (iTLC_SA_, *R*_f_ protein = 0, *R*_f_ [^99m^Tc(CO)_3_]^+^ = 1) and analytical HPLC SEC (BioSep SEC-s2000, Phenomenex). Protein concentration after gel filtration was determined by UV absorption after decay using an aliquot stored at −80°C.

### Cell binding studies

Binding properties of the radiolabelled diabody were analysed in homologous competition studies. Cells (5 × 10^4^ DU145 or DU145-PSMA cells/well) were seeded in 96-well plates, grown overnight and incubated the next day with serial dilutions of J591Cdia (1,850 to 0.03 nM) and a constant concentration of ^99m^Tc-labelled diabody (1 nM) at 4°C for 45 min. Cells were washed with 3 × 100 μl cold PBS and lysed with 2 × 50 μl 0.5 M NaOH. Cell-associated activity was measured by gamma counting (1282 Compugamma Universal Gamma Counter, LKB Wallac, PerkinElmer, Cambridge, UK). Data were analysed with GraphPad Prism and fitted using a ‘one-site total binding’ algorithm. *K*_d_ was calculated as IC_50_ − [^99m^Tc-J591Cdia].

### Serum stability studies

The diabody was labelled with ^99m^Tc as described above, mixed 1/1 with fresh human serum and incubated at 37°C. Samples were taken at 0, 15, 30, 60, 120 and 240 min and analysed by thin-layer chromatography using iTLC_SA_ chromatography paper (Agilent Technologies, Wokingham, UK) and 100 mM citrate buffer (pH 5.2) as mobile phase. To discriminate serum-bound and antibody-bound activity, samples taken at the same time points were frozen in liquid nitrogen, separated by SDS-PAGE (12% NuPAGE, Invitrogen, Paisley, UK) and analysed by autoradiography (Cyclone Plus, PerkinElmer, Waltham, USA).

### Subcutaneous prostate cancer xenograft model

Animal studies were carried out in accordance with UK Research Councils' and Medical Research Charities' guidelines on Responsibility in the Use of Animals in Bioscience Research, under UK Home Office Project and Personal licences. Male SCID beige mice aged 6 to 10 weeks (Charles River, Margate, UK) were used for all experiments. Cells (3 to 4 × 10^6^ DU145 or DU145-PSMA cells) were injected subcutaneously on the left flank in 50 μl RPMI 1640 medium, and animals were used for imaging or biodistribution studies after 4 to 5 weeks, when tumours reached approximately 5 mm in diameter. The DU145/DU145-PSMA model was used instead of the commonly used LNCaP/PC3 combination of tumours to take advantage of the more reproducible growth of the xenografts. The use of the same cell line with and without target receptor expression should further allow a more accurate evaluation of specific versus unspecific uptake in the tumour tissue.

### Biodistribution studies

Labelled diabody (J591Cdia-[^99m^Tc(CO)_3_]^+^, 10 to 13 μg) was injected via the tail vein, and mice were sacrificed after 8 h (four mice/group). Organs were dissected, briefly washed in PBS, blotted dry and weighed. Activity in whole organs and tumours was measured by gamma counting and is expressed as percent injected dose (ID)/gram. The total injected dose was determined by weighing the syringes before and after injection and referring to serial dilutions of the labelled antibody, prepared from a separate syringe and measured along with the dissected organs.

### *In vivo* SPECT imaging

Single photon emission tomography was performed with a small-animal SPECT/CT scanner (Mediso, Budapest, Hungary) under isofluorane anaesthesia and respiration monitoring. Mice (three to four mice/group) were injected via the tail vein with 25 to 35 MBq of labelled diabody (10 to 13 μg, 0.22 to 0.24 nmol in 50 to 80 μl PBS), and helical SPECT/CT images were acquired at 0, 20 and 40 min, and again at 4 and 8 h post injection (with 15, 30 and 45 min of acquisition time). CT images were acquired after each SPECT scan.

### Image analysis

SPECT images were reconstructed with HiSPECT™ software (Bioscan, Washington, DC, USA). CT images were reconstructed using the SPECT/CT scanner-embedded software package. Maximum intensity projection (MIP) images were generated and scaled individually. To quantify tumour and muscle uptake, tissues were identified in the CT images and regions of interest covering the entire tumour were drawn in all slices acquired. Relative uptake values were used to generate time-activity curves for individual tumours.

### Immunofluorescence staining of tumour sections

For *ex vivo* validation of PSMA expression in individual tumours, tissue was harvested at the end of an experiment and frozen in liquid nitrogen. Cryo-sections (10 μm) were prepared and stained with the AlexaFluor-488-labelled version of the J591C diabody. Briefly, air-dried sections were fixed with 4% formalin, washed with PBS and incubated with 1.4 μg/ml labelled diabody in PBS/1% BSA for 45 min at RT. Sections were washed with PBS, slides were mounted with mounting media containing DAPI to counterstain nuclei (ProLong Gold, Invitrogen) and images were taken with a TCS SP5 II confocal microscope (Leica, Wetzlar, Germany).

### Statistical analysis

Unless otherwise stated, values are shown as mean ± standard deviation (SD). Data were analysed for statistical significance using GraphPad Prism version 5.00 for Windows. Unless otherwise stated, an unpaired, two-tailed *t* test was used. Statistical significance was assigned for *p* values <0.05.

## Results

### Diabody expression, purification and *in vitro* characterisation

**Figure 1:**
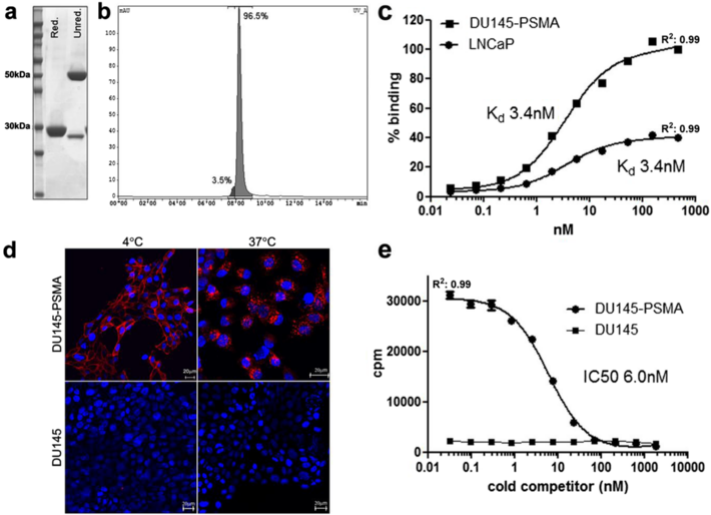
***In vitro *characterisation of J591C diabody. (a)** SDS-PAGE of purified J591C diabody. The reduced sample runs as a single band corresponding to the size of the monomer (27 kDa). In the non-reduced sample, the majority of protein is present as a covalent dimer through formation of a disulfide bond by the C-terminal cysteines. **(b)** Analytical size exclusion HPLC. The UV chromatogram shows elution as a single dimeric species. **(c)** Saturation binding assay on DU145-PSMA and LNCaP cells with J591Cdia-AlexaFluor-488, labelled via the C-terminal cysteines and analysed by flow cytometry. DU145-PSMA cells show higher PSMA expression levels than LNCaP. **(d)** Binding and internalisation studies with the AlexaFluor-488-labelled diabody in DU145-PSMA and DU145 cells at 4°C and 37°C (20 min of incubation). **(e)** Competitive binding assay with 1 nM [^99m^Tc(CO)_3_]^+^-labelled diabody vs. unlabelled diabody as cold competitor on DU145-PSMA and DU145 cells (data representative of four independent experiments).

The diabody was produced in HEK293T cells with yields of purified protein (purity >95%) of 4 to 6 mg/L culture supernatant. The diabody was present as a dimer of 54 kDa with the C-terminal cysteines forming a disulfide bond in 80% to 90% of the protein, as shown by SDS-PAGE analysis of reduced and unreduced samples (Figure 1a). Dimer formation does not depend on the C-terminal cysteine. When expressed without the cysteine, the diabody (J591dia), as the version with cysteine, elutes as a dimer in size exclusion HPLC. In both cases, no monomeric diabody was detected during purification or subsequent size exclusion HPLC analyses. Binding properties of the J591Cdia were analysed by flow cytometry and confocal microscopy. For this purpose, the protein was labelled with Alexa488-maleimide or fluorescein maleimide via its C-terminal cysteine. In saturation binding assays, the diabody showed specific binding to PSMA^+^ cells with a *K*_d_ of 3.3 ± 0.2 nM (mean of four independent experiments).The diabody is internalised rapidly upon incubation with DU145-PSMA cells as determined by confocal microscopy. After 20 min at 37°C, all labelled diabody was detected in intracellular vesicles while at 4°C mostly membrane staining was observed. No binding was detected on DU145 cells (Figure 1d).

To enable SPECT imaging, J591Cdia was radiolabelled by site-specific chelation of [^99m^Tc(CO)_3_]^+^ by the C-terminal (His)_6_-tag. The labelling method consistently yielded >85% labelling after 1 h and >95% after 2 h of incubation at 37°C (Additional file 2). After 1 h of incubation, the labelled diabody was separated from unbound activity by a gel filtration step resulting in a final radiochemical purity of >99% (Additional file 2) and a corresponding specific activity of 4.2 MBq/μg (231 MBq/nmol diabody).

Stability of the radiolabel upon incubation in human serum was analysed by iTLC_SA_ and SDS-PAGE. No loss of radiolabel was observed over a period of 4 h at 37°C (Additional file 2). Similarly, no activity was released upon incubation in PBS (data not shown), and no significant *trans*-chelation of [^99m^Tc(CO)_3_]^+^ to serum proteins was observed (Additional file 2).

Competitive binding studies with ^99m^Tc-labelled vs. unlabelled J591Cdia confirmed specific binding to PSMA^+^ cells with a *K*_d_ of 5.0 nM ± 0.5 (*n* = 4; Figure 1e).

### DU145/DU145-PSMA tumour model

**Figure 2:**
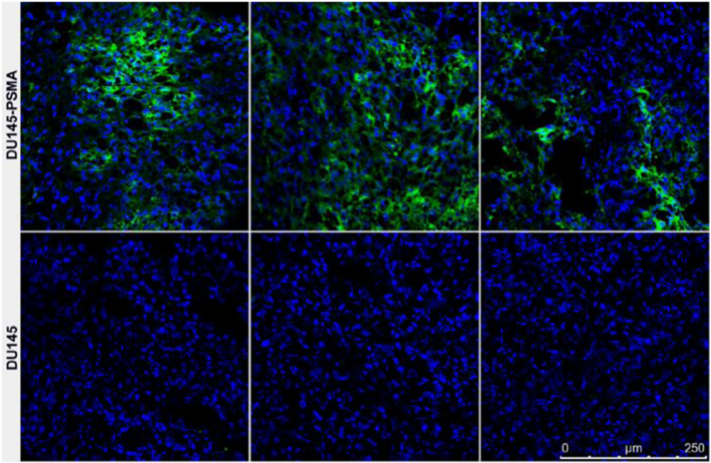
***Ex vivo *verification of PSMA expression in DU145-PSMA and DU145 tumours.** Immunofluorescence staining of tumour cryo-sections was performed using J591Cdia-AlexaFluor-488 (green). Nuclei are counterstained with DAPI (blue). Three representative tumour sections are shown. All analysed DU145-PSMA sections showed strong, heterogeneous staining for PSMA. No staining was observed in DU145 tumour sections.

LNCaP cells were used as a reference for the amount of PSMA expressed by the transfected DU145 cells. *B*_max_ values obtained from saturation binding studies were used to compare expression levels, showing an approximately 2.4 times higher number of receptors on DU145-PSMA cells (Figure 1c). The tumour take rate (tumours of >3 × 3 mm at 4 weeks post injection (p.i.)) was >80% for both DU145 and DU145-PSMA cells (*n* = 46). PSMA expression in the PCa xenografts was determined *ex vivo* by immunofluorescence staining of tumour sections (Figure 2). Cryo-sections were stained using the diabody conjugated to AlexaFluor-488. In agreement with flow cytometry data using the DU145 and DU145-PSMA cell lines (not shown), a heterogeneous expression pattern was observed in all DU145-PSMA tumour sections while staining was absent from DU145 tumours.

### Biodistribution and SPECT imaging

**Figure 3:**
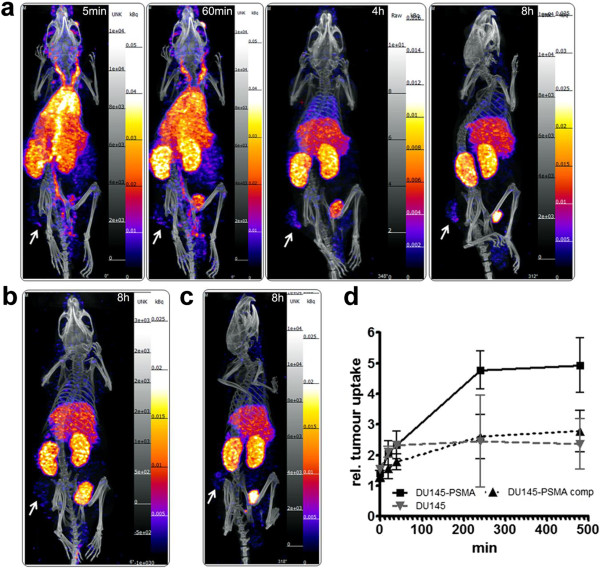
**MIP images of SPECT/CT scans with **^**99m**^**Tc-J591Cdia in mice bearing subcutaneous prostate carcinoma tumours.** Mice with established DU145-PSMA or control DU145 tumours received 10 to 13 μg (between 25 and 35 MBq/injection) of ^99m^Tc-J591Cdia by tail vein injection. **(a)** Serial images at 5 min, 60 min, 4 h and 8 h post injection of a mouse with DU145-PSMA tumour. **(b)** Mouse with PSMA-negative DU145 tumour at 8 h p.i. **(c)** Mouse with DU145-PSMA tumour; binding of ^99m^Tc-J591Cdia blocked with a 20 times excess of unlabelled diabody (cold competition, at 8 h p.i.). **(d)** Tumour uptake values in the DU145-PSMA (*n* = 3), DU145 (*n* = 3) and cold competition (comp, *n* = 4) groups over time as determined from SPECT/CT images (shown as mean ± SD). Arrows indicate the position of the tumour in each image. Images are scaled individually.

Targeting properties of the [^99m^Tc(CO)_3_]^+^-labelled J591Cdia were assessed by serial SPECT/CT imaging of tumour-bearing mice from 20 min to 8 h p.i. and by *ex vivo* biodistribution studies. The serial images shown in Figure 3a demonstrate that J591Cdia was initially observed in the blood pool (at 0 to 60 min), followed by a progressive increase in uptake in the liver, kidneys and bladder, owing to urinary excretion (4 and 8 h time points). No excretion via the hepato-biliary route was observed.

The diabody accumulated specifically in PSMA^+^ tumours with a clear delineation of the tumour observed from 4 h post injection. Tumour uptake values obtained from the images (three to four mice/group) show the highest accumulation of the tracer at 8 h p.i. and slightly lower uptake at 4 h p.i. (Figure 3d). The ratio of PSMA-specific vs. unspecific accumulation (DU145-PSMA/DU145 tumour) was 1.8 (4 h) and 2.1 (8 h), respectively. Injection of a 20-fold excess of unlabelled diabody reduced DU145-PSMA tumour uptake to the same level observed in the PSMA-negative DU145 tumours.

**Figure 4:**
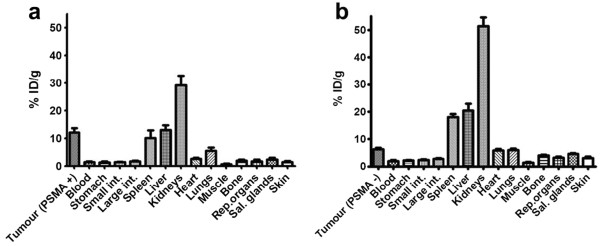
***Ex vivo *biodistribution of **^**99m**^**Tc-J591Cdia in mice with subcutaneous DU145 or DU145-PSMA tumours p.i. (a)** DU145-PSMA tumour. **(b)** DU145 tumour. As for the imaging studies, mice (*n* = 4) with established tumours received 10 to 13 μg (between 25 and 35 MBq/injection) of ^99m^Tc-J591Cdia by tail vein injection. Tracer uptake in selected organs at 8 h p.i., as measured by gamma counting, is expressed as mean percent injected dose/gram.

Results from the *ex vivo* biodistribution study are shown in Figure 4 (and Table 1). The diabody accumulated in the tumour with 12.1% ± 1.7% ID/g at 8 h p.i. High uptake was also observed in the kidneys (29.2% ± 1.7% ID/g), liver (13% ± 0.9% ID/g) and spleen (10.1% ± 1.4% ID/g). The tumour to blood and tumour to muscle ratios at 8 h p.i. were 8.0 and 16.8, respectively. The tumour uptake in the DU145 control group was significantly lower, with 6.3% ± 0.5% at 8 h p.i. (*p* < 0.001) and a PSMA^+^ to PSMA^−^ ratio of 1.9. The tumour to blood and tumour to muscle ratios were 3.3 and 4.8, respectively. Unexpectedly, higher activity was observed in several organs in the DU145 group compared to the DU145-PSMA group (44% injected dose (ID) vs. 67% ID in all organs at 8 h p.i.). The average tumour weight was 66 ± 14.6 mg for DU145-PSMA and 80 ± 24.5 mg for DU145 (no significant difference in weight, *p* > 0.5).

**Table 1:** ***Ex vivo *** **biodistribution of **^**99m**^**Tc-J591Cdia at 8 h post injection**

**Organ**	**DU145-PSMA**	**DU145**
Tumour	12.1 ± 1.7	6.3 ± 0.5
Blood	1.5 ± 0.2	1.9 ± 0.4
Stomach	1.4 ± 0.3	2.2 ± 0.1
Small intestine	1.4 ± 0.2	2.4 ± 0.2
Large intestine	1.7 ± 0.3	2.7 ± 0.3
Spleen	10.1 ± 2.8	18.0 ± 1.2
Liver	13.0 ± 1.8	20.5 ± 2.5
Kidneys	29.2 ± 3.5	51.4 ± 3.2
Heart	2.7 ± 0.3	5.8 ± 0.5
Lungs	5.5 ± 1.2	6.0 ± 0.6
Muscle	0.7 ± 0.2	1.3 ± 0.3
Bone	2.0 ± 0.4	4.0 ± 0.4
Reproductive organs	1.8 ± 0.7	3.2 ± 0.4
Salivary glands	2.4 ± 0.7	4.5 ± 0.4
Skin	1.4 ± 0.4	3.1 ± 0.5
Tumour to blood ratio	8	3.3
Tumour to muscle ratio	16.8	4.8
Ratio of DU145-PSMA to DU145 tumour uptake	1.9	-

*Ex vivo* biodistribution of ^99m^Tc-J591Cdia in mice with subcutaneous DU145 or DU145-PSMA tumours at 8 h post injection (*n* = 4). Values are expressed as mean percent injected dose per gram of tissue (% ID/g) ± SD.

## Discussion

A J591-derived diabody was developed, labelled with ^99m^Tc and characterised as a SPECT tracer for imaging of PSMA expression in prostate cancer. The use of a diabody combines the specificity and targeting properties of the parental antibody with more favourable pharmacokinetics for imaging purposes.

The J591C diabody (J591Cdia) was produced as a stable dimer with good yields obtained by batch expression in HEK293T cells. The use of C-terminal cysteines to promote formation of covalent dimers and for the purpose of site-specific modification has been described previously. In accordance with published data, we observed the formation of an inter-chain disulfide bond in up to 90% of the purified protein [30,31]. Both versions of the J591C diabody, either fluorescently labelled via the C-terminal cysteines or radiolabelled with intact inter-chain disulfide bond, bound to PSMA-expressing cells with low nanomolar affinity. The chelation of [^99m^Tc(CO)_3_]^+^ by genetically encoded histidine tags offers a simple yet site-specific and stable means to label proteins with ^99m^Tc [27,32], obviating the additional step of conjugating a chelator to the protein. For the J591C diabody, this labelling method proved robust, allowing for reproducible labelling to high specific activity.

Antibody fragment-based radiotracers should allow earlier image acquisition as compared to their full-length antibody counterparts, without compromising the sensitivity and specificity of detection. In clinical studies with ^111^In- or ^177^Lu-labelled versions of the parental full-length antibody hJ591, a serum half-life (*T*_1/2_ β) of 44 ± 15 h was reported and clear images were obtained as late as 4 to 7 days post injection [33]. Preclinical evaluation of a ^89^Zr-desferrioximine (DFO)-huJ591 conjugate showed a plateau in uptake in LNCaP tumours from about 48 h p.i. [34]. By contrast, the *T*_1/2_ β of diabody molecules in mice has been reported as between 1 and 7 h [21,23,35,36], and 4 to 12 h post injection has been suggested as the optimal time point for diabody imaging [20]. In keeping with this, the J591C diabody shows a plateau in accumulation in the tumour from about 4 h p.i. and good tumour to blood and tumour to muscle ratios at 8 h p.i. (8 and 16.8). An excess of cold diabody could block tumour uptake efficiently. The elevated uptake in DU145 tumours (lacking PSMA) is presumably a result of the enhanced permeation and retention (EPR) effect, which is commonly found in well-perfused tumours and with molecules of this size.

The liver uptake observed in our study is moderate (13% ± 0.9% ID/g) and comparable to that found for the full-length 89Zr-(DFO)-huJ591 [34]. Whether this partial hepatic clearance interferes with the detection of potential metastases in the liver will have to be investigated. Since the choice of the radiolabel can influence uptake and retention in target and non-target tissues [37], alternative labels may be compared in follow-up studies.

We observed a higher activity in several organs in the DU145 compared to the DU145-PSMA group. The reason for this is unclear; a possible explanation may be a slower initial renal filtration of the tracer and a resulting higher uptake in susceptible organs in the different batch of mice.

The extent of kidney uptake and retention suggests binding of J591Cdia to PSMA expressed in the renal proximal tubules. This has been observed similarly in preclinical studies with small-molecule inhibitors targeting PSMA [38,39]. Renal excretion can potentially interfere with the detection of small lesions or recurrent disease in the prostate bed, although this has not been reported to be a prominent issue in clinical studies with the small PSMA inhibitors [9,10].

Site-specific PEGylation has been shown to gradually reduce renal filtration of a diabody with increasing PEG size and increased tumour uptake as a result of the longer circulation time [22]. This strategy could potentially be adopted for the J591Cdia, however at the cost of lower contrast at early time points.

Several urea-based inhibitors of the glutamate carboxypeptidase activity of PSMA have been described and developed as diagnostic tracers. Recently, promising results of phase 1 clinical trials with two ^123^I-labelled compounds and a ^68^Ga-labelled molecule were reported [9,10]. A ^99m^Tc-labelled version showed excellent biodistribution properties preclinically with rapid renal clearance and tumour to blood and tumour to muscle ratios of 550 and 157, respectively, at 4 h p.i. [38]. This compound is currently in a phase II trial (ClinicalTrials.gov Identifier: NCT01667536).

Comparison of these small-molecule tracers with the results of two ongoing PCa imaging trials with ^89^Zr-(DFO)-huJ591 (ClinicalTrials.gov Identifier: NCT01543659) and a ^89^Zr-labelled minibody derivative (ClinicalTrials.gov Identifier: NCT01923727) in terms of their sensitivity and specificity may indicate the future potential of antibody-based imaging of PSMA.

## Conclusion

In conclusion, a diabody derived from the well-established anti-PSMA antibody J591 was engineered and shown to specifically target PSMA-positive tumours allowing for SPECT imaging with high contrast from 4 to 8 h post injection.

## Competing interests

The authors declare that they have no competing interests.

## Authors' contributions

FK designed the study, carried out the experiments and wrote the manuscript. JW carried out biodistribution and protein labelling experiments. JM carried out experiments and revised the manuscript. GEM and PJB revised the manuscript. All authors read and approved the final manuscript.

## Supplementary Material

Additional file 1 Schematic of a full-length antibody and a diabody with C-terminal cysteine. Click here for file

Additional file 2 **Radiolabelling of J591C diabody with [**^**99m**^**Tc(CO)**_**3**_**]**^**+**^. Figure showing the labelling of J591Cdia with [^99m^Tc(CO)_3_]^+^ over time as measured by TLC and analysis of serum stability of ^99m^Tc-J591Cdia as measured by TLC and SDS-PAGE. Click here for file
