# Supplementary material for: Design and preclinical evaluation of a 99mTc-labelled diabody of mAb J591 for SPECT imaging of prostate-specific membrane antigen (PSMA)
Source: EJNMMI Res. 2014 Mar 7;4:13. doi: 10.1186/2191-219X-4-13 (PMC4015168; doi:10.1186/2191-219X-4-13)
Supplement: Additional file 1 — Schematic of a full-length antibody and a diabody with C-terminal cysteine. [file 2191-219X-4-13-S1.docx]

**Additional file 1**

**Design and preclinical evaluation of a Tc-99m-labelled diabody of mAb J591 for SPECT imaging of prostate specific membrane antigen (PSMA)**

Kampmeier, F. ^1*^, Williams, J.^1^, Maher, J. ^2,3,4^, Mullen, G.E.^1^, Blower, P.J.^1^

**
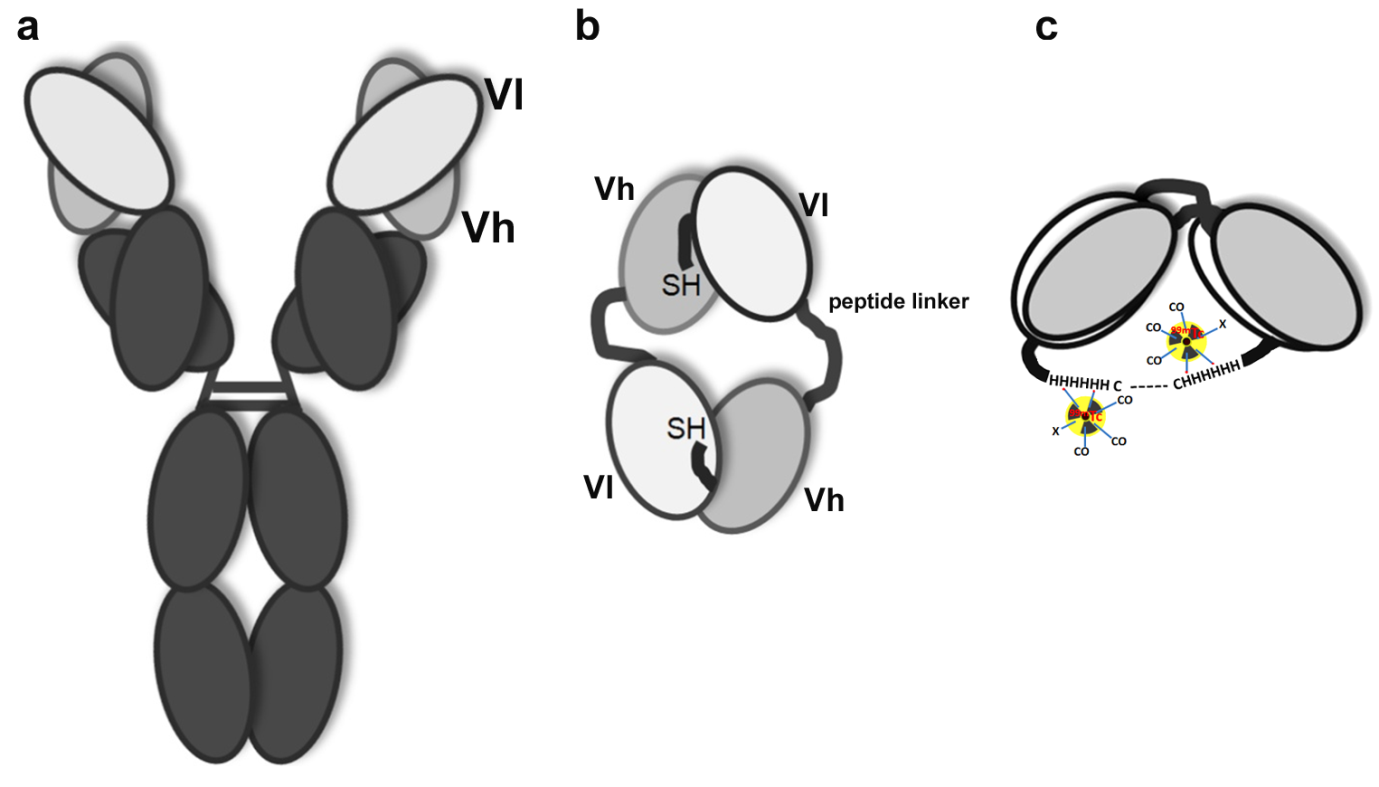
**

Schematic of a full-length antibody and a diabody with C-terminal cysteine

The diabody consists of only the variable, antigen-binding domains of the parental antibody, connected by a short peptide linker, resulting in formation of a dimeric bivalent derivative. Addition of a C-terminal cysteine results in formation of an inter-chain disulfide bond that can further stabilise the dimerisation. a) Full length IgG antibody molecule (~150kDa), b) diabody with C-terminal cysteine (~55kDa), c) diabody with C-terminal cysteines forming an inter-chain disulfide bond and with bound [^99m^Tc(CO)_3_]^+^. Vh: Variable domain heavy chain, Vl: Variable domain light chain.
