# Supplementary material for: Design and preclinical evaluation of a 99mTc-labelled diabody of mAb J591 for SPECT imaging of prostate-specific membrane antigen (PSMA)
Source: EJNMMI Res. 2014 Mar 7;4:13. doi: 10.1186/2191-219X-4-13 (PMC4015168; doi:10.1186/2191-219X-4-13)
Supplement: Additional file 2 — Radiolabelling of J591C diabody with [99mTc(CO)3]+. Figure showing the labelling of J591Cdia with [99mTc(CO)3]+ over time as measured by TLC and analysis of serum stability of 99mTc-J591Cdia as measured by TLC and SDS-PAGE. [file 2191-219X-4-13-S2.docx]

**Additional file 2**

**Design and preclinical evaluation of a Tc-99m-labelled diabody of mAb J591 for SPECT imaging of prostate specific membrane antigen (PSMA)**

Kampmeier, F. ^1*^, Williams, J.^1^, Maher, J. ^2,3,4^, Mullen, G.E.^1^, Blower, P.J.^1^


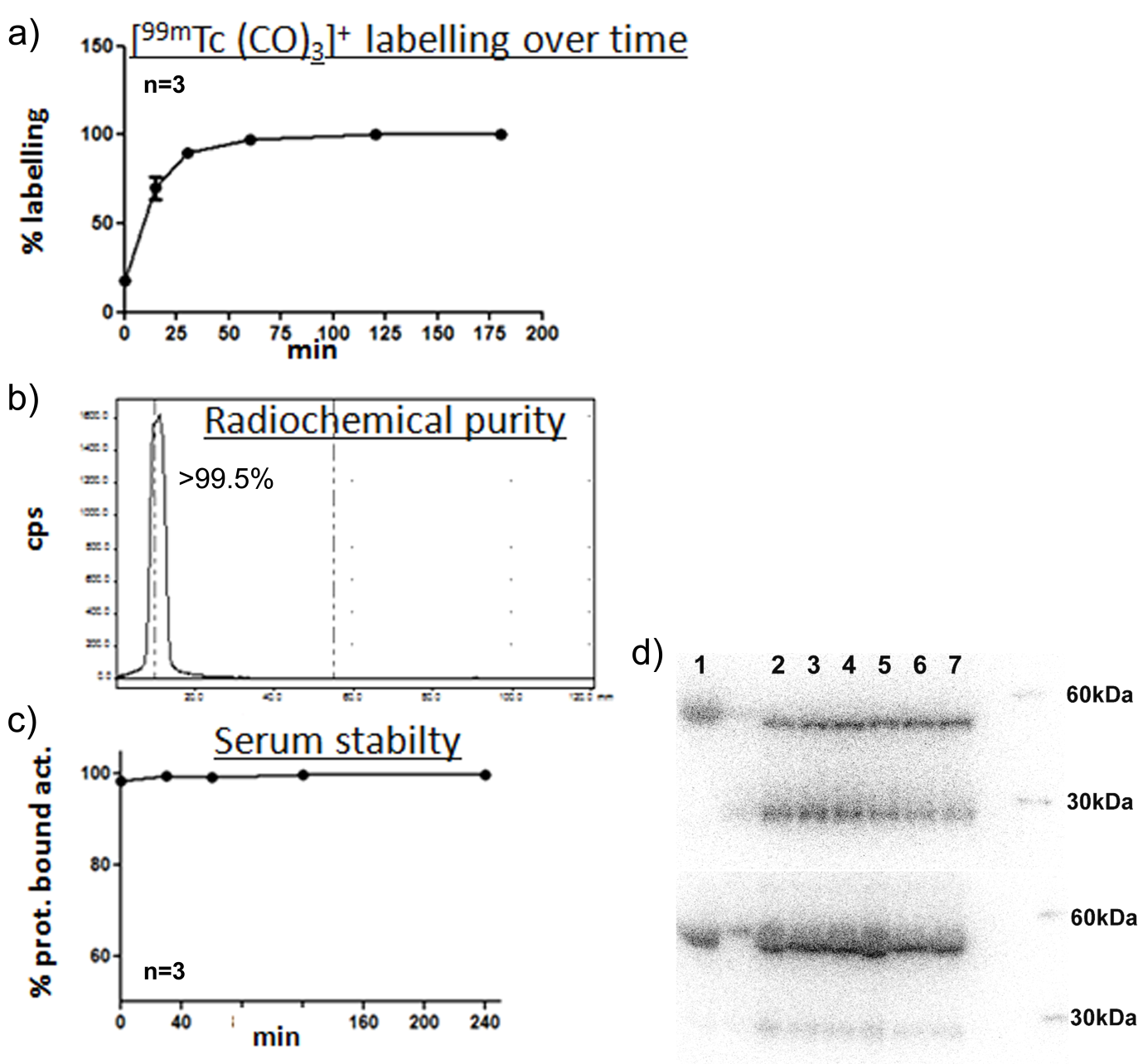


**Radiolabelling of J591C diabody with [^99m^Tc(CO)_3_]^+^**

a) Radiolabelling over time, measured by thin layer chromatography (TLC).

b) Radiochemical purity of J591Cdia-^99m^Tc after G25 Minitrap purification, measured by TLC.

c) Stability of the ^99m^Tc radiolabel in human serum at 37°C measured by TLC.

d) Trans-chelation of [^99m^Tc(CO)_3_]^+^ to serum protein measured by SDS-PAGE in partially reduced (top) and unreduced samples (bottom) (1: Serum + [^99m^Tc(CO)_3_]^+^, 240min, 2-7: J591Cdia-^99m^Tc + serum at 0, 15, 30, 60, 120 and 240min). Comparison of partially reduced and unreduced samples shows association of [^99m^Tc(CO)_3_]^+^ with the diabody molecule (dimer: 54kDa, monomer 27kDa).
